# Chemical Characterization, Nutritional and Bioactive Properties of *Physalis peruviana* Fruit from High Areas of the Atacama Desert

**DOI:** 10.3390/foods10112699

**Published:** 2021-11-04

**Authors:** Patricio Muñoz, Felipe Parra, Mario J. Simirgiotis, Germán F. Sepúlveda Chavera, Claudio Parra

**Affiliations:** 1 Laboratorio de Patología Vegetal y Bioproductos, Facultad de Ciencias Agronómicas, Universidad de Tarapacá, Av. General Velásquez 1775, Arica 1000000, Chile; pmunozt@ucdavis.edu; 2 University of California Davis Chile Life Sciences Innovation Center, Av. Santa María 2670, Santiago 7520424, Chile; 3 Laboratorio de Química Orgánica y Productos Naturales, Facultad de Ciencias Agronómicas, Universidad de Tarapacá, Av. General Velásquez 1775, Arica 1000000, Chile; filyparramontes@hotmail.com; 4 Instituto de Farmacia, Facultad de Ciencias, Universidad Austral de Chile, Valdivia 509000, Chile; mario.simirgiotis@uach.cl

**Keywords:** antioxidant, antibacterial, Atacama Desert, Cape gooseberry, enzyme inhibition, UHPLC-DAD

## Abstract

*Physalis peruviana* L. belongs to the Solanaceae family and produces a spherical fruit used to treat various diseases. However, the chemical composition, nutritional characterization, and bioactive properties of the *P. peruviana* growing in the Andean region of the Atacama Desert have not been conducted so far. The results showed clear differences in the nutritional and bioactive characteristics of the fruits grown in arid environmental conditions, which were comparable to those from countries with a production tradition. The fruits studied showed a higher Ca, Cu, Mn, P, and Zn content and bioactive compounds such as flavonoids and tannins than those reported in the literature. UHPLC was performed to determine the main phenols. Gallic acid was identified as the predominant phenolic compound in this species (303.63 mg/100 g FW), of which to our knowledge no previous study has reported similar concentrations in this species. Moreover, Cape gooseberry extract has antioxidant and antimicrobial activity against Gram-positive and Gram-negative bacteria. *Pseudomonas syringae* (MIC 0.313 mg/mL and MBC 1.25 mg/mL) was the most susceptible bacterium. Meanwhile, *Erwinia rhapontici* was the most resistant bacterium (MIC and MIB 5.00 mg/mL). Furthermore, it was found to inhibit α-amylase activity with an IC_50_ value (39.28 µg/mL) similar to that of acarbose (35.74 µg/mL). These results expand the knowledge of the species cultivated in arid environmental conditions and suggest an alternative for the potential use of this fruit to manage chronic diseases such as diabetes.

## 1. Introduction

The leading causes of death in humans are cancer and cardiovascular diseases, and oxidative stress is believed to be a contributing factor in the development of these diseases [1]. The antioxidant properties of phytochemicals, found especially in fruits and vegetables, may help to avoid oxidative stress on the body [2,3]. Cape gooseberry (*Physalis peruviana*; PP) is a potential candidate for elaborating original functional foods because of its nutritional properties and biologically active components. In particular, the pulp is a good source of provitamin A, vitamin C, vitamin B complex, phenolic compounds, and various minerals of nutritional importance [4,5,6,7]. The chemical composition of *P. peruviana* fruit extract has indicated the presence of different chemical compounds, such as saponins, withanolides, peruvioses, irinians, kaempferol, and quercetin di- and tri-glycosides [8,9,10], some of which have demonstrated antioxidant [11], hypoglycemic [12], and anticancer activities [13,14]. Although golden berries are generally commercialized as fresh products, the fruits are also used in sauces, syrups, and marmalades or dehydrated (similar to grape raisins) for bakeries, cocktails, snacks, and breakfast cereals [13,15].

**Figure 1:**
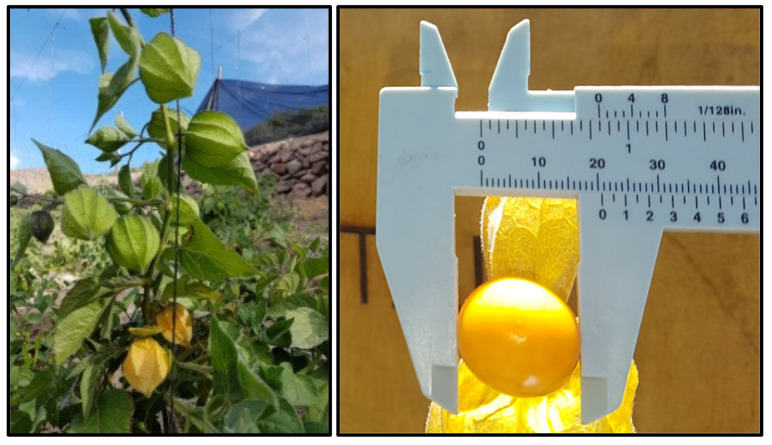
*Physalis peruviana* fruit from the Arid Andean Region of Northern Chile.

In this context, a diverse diet based on fruits and vegetables greatly benefits human health and prevents chronic diseases. One of these fruits with healthy properties is the fruit of the Cape gooseberry; however, it is underused in the current food production system, and its nutritional benefits are vastly underutilized. This crop is associated with high-altitude areas of South America, with its origin located in the Peruvian Andes [16]. However, it is found in almost all highlands of the tropics and at several sites in the subtropics, including Malaysia, China, Africa, and the Caribbean [17]. Colombia is the primary producer of Cape gooseberry, with around 90% of the total world production; it is also cultivated on a smaller scale in South Africa, Ecuador, Peru, Zimbabwe, and Mexico; and minor experiences have occurred in Spain, France, southern Italy, Australia, India, the United Kingdom and Kenya [3]. Recently, in Chile, this crop was cultivated in the Andean region of the Atacama Desert, producing a spherical fruit of intense color with many seeds (Figure 1) [18]. Another study reported the nutritional and bioactive properties of cultivated and wild fruits of *P. peruviana* growing in the northern Argentinean area [4]. However, the chemical composition and biological activities present in the fruits grown in the Atacama Desert have not yet been characterized. For this reason, this work aims to evaluate the enzyme inhibition and antioxidant and antimicrobial activities of Cape gooseberry grown in the Andean Region of the Atacama Desert in northern Chile and its phenolic composition.

## 2. Materials and Methods

### 2.1. Fruit and Sample Preparation

*P. peruviana* was harvested from cultivated plants in the Andes of northern Chile (latitude S 18°33.222′0″; longitude W 69°29.513′0″, 3317 m.a.s.l). According to the visual analysis, the samples were homogeneously selected according to color, size, and maturity. The Cape gooseberry samples were dried in a freeze-dryer (Virtis Benchtop model, 3L, Gardiner, NY, USA) for about 48 h with the condenser temperature and chamber vacuum at −50 °C and 12.5 Pa, respectively. The fruits were cleaned and washed underwater, and then it was freeze-dried and ground into a fine powder using an electric blender. The dried and powdered sample (500 mg) was macerated with absolute ethanol for 72 h at room temperature. The macerate was filtered and concentrated under reduced pressure.

### 2.2. Chemicals

HPLC standards, apigenin, caffeic acid, chlorogenic acid, *trans*-cinnamic acid, ferulic acid, gallic acid, kaempferol, luteolin, naringenin, *p*-coumaric acid, vanillic acid, and 4-hydroxybenzoic acid were purchased from Sigma Aldrich (Santiago, Chile). Aluminum chloride hexahydrate, anhydrous sodium carbonate, ethanol absolute, formic acid, ferric chloride hexahydrate, Folin–Ciocalteu reagent, hydrochloric acid, HPLC solvents, sodium hydroxide, sodium nitrate, and sodium-potassium tartrate were obtained from Merck (Santiago, Chile). Alpha-amylase from porcine pancreas, 3,5-dinitrosalicylic acid, 4-nitrophenyl-α-D-glucopyranoside, alpha-glucosidase from *Saccharomyces cerevisiae*, ABTS (2,2′-azinobis (3-ethylbenzothiazoline-6-sulphonate)), acarbose, glacial acetic acid, potassium persulfate, starch, TPTZ (1,3,5-triphenyltetrazolium chloride), and Trolox (6-hydroxy-2,5,7,8-tetramethylchroman-2-carboxylic acid) were supplied by Sigma-Aldrich (Santiago, Chile).

### 2.3. Determination of Proximate Composition

AOAC procedures were used in all determinations [19]. The water content was determined by oven-drying samples at 105 °C to a constant weight. Crude ash content was estimated by incineration in a muffle furnace at 550 °C. Total lipids were determined by extracting a known weight of powdered samples with diethyl ether using a Soxhlet apparatus. Crude protein content was realized by means of the Kjeldahl procedure, using a conversion factor of 6.25. The samples were digested using a DK-6 digester and distilled using a UDK 129 distilling unit (VELP Scientifica, Usmate Velate, Italy). The crude fiber content of the sample was determined via the acid/alkaline hydrolysis of fat-free samples. Total carbohydrates were calculated by difference.

### 2.4. Determination of Mineral Content

The mineral content in the Cape gooseberry was determined with the ash sample previously described. The ash sample was dissolved in 5 mL of HNO_3_ (50%) solution and heated on a hotplate until complete digestion. The sodium, potassium, calcium, magnesium, manganesium, iron, copper, and zinc concentrations were determined using an atomic absorption spectrophotometer (AA240, Varian Inc., Palo Alto, CA, USA).

### 2.5. Instrument Conditions for Phenolic Compounds

The method described by Soto et al. [20], with some modifications, was used. The Knauer Azura analytical UHPLC system (Knauer, Berlin, Germany) was equipped with an Azura DAD 2.1 L diode array detector with high-sensitivity Knauer 3950 autosamplers, and an Azura P 6.1 L pump operated at 25 °C. A reversed-phase column (Purospher^®^ STAR RP-18 end capped, 150 mm × 4.6 mm, 3.0 µm, Merck, Darmstadt, Germany) was used. The separation was achieved using (A) 1% formic acid in the water, and (B) acetonitrile as mobile phase at 0.7 mL/min with a gradient: at 0 min, the A:B ratio was 95:5; at 6 min, 70:30; at 16 min, 50:50; at 26 min, 30:70; at 36 min, 5:95; and at 46 min, 95:5. The system was allowed to run for another 10 min to equilibrate the column before each injection. The most likely identification of phenolics was achieved by comparing the retention times and HPLC spectra of each peak in the sample with those of the respective phenolic compound standards.

### 2.6. Phytochemical Analysis

#### 2.6.1. Phenolic and Flavonoid Contents

Total phenol content was determined using the Folin–Ciocalteau method, adapted to a 96-well microplate [21]. Each sample was measured at 760 nm and compared to a reference standard calibration curve. Results were reported as gallic acid equivalents per 100 g on fresh weight (mg GAE/100 g FW). The total flavonoid content was estimated as suggested by Echiburu-Chau et al. using the aluminum chloride colorimetric method [22]. The samples were measured at 415 nm and compared to a calibration curve using quercetin as the standard. Measurements were expressed in mg QE/100 g FW.

#### 2.6.2. Total Tannin Content

Total tannin content was measured using the Folin–Denis method described by Belwal et al. [23] with slight modifications. Briefly, 250 µL of Folin–Denis reagent was mixed properly with 2500 µL of stock solution. Then, 500 µL of sodium carbonate (7% *v*/*v*) and 1750 µL of distilled water were added to this mixture and kept for 20 min at room temperature. Each sample was measured at 700 nm and compared to a calibration curve using tannic acid as the standard.

#### 2.6.3. Total Anthocyanin Content

Total anthocyanin content was measured per the AOAC pH differential method, as described by El-Beltagi et al. [13]. According to this method, 250 µL of stock solution was diluted with potassium chloride (0.025 M) to pH 1.0 and measured at 520 nm. Similarly, 250 µL of stock solution was diluted with sodium acetate (0.400 M) to obtain a pH of 4.5 and measured at 700 nm. Both samples were to incubate for 20 min at room temperature. Absorbance was measured at 520 and 700 nm and the absorbance values were found using Equation (1):

(1) $$A=\;\left({\mathrm{Abs}}_{520}\;-\;{\mathrm{Abs}}_{700}\right)\mathrm{pH}1-\;\left({\mathrm{Abs}}_{520}\;-\;{\mathrm{Abs}}_{700}\right)\;\mathrm{pH}4.5$$

The total amount of anthocyanin was calculated as follows:

$$\mathrm{TA}\;\left(\mathrm{mg}\;\mathrm{CGE}/100\;g\;\mathrm{FW}\right)\;=\frac{\left(A\times \mathrm{MW}\times \mathrm{df}\times 10^{3}\times 100\right)}{\left(\varepsilon \times 1\right)}$$

where MW = molecular weight (449.2 g/mol of cyanidin 3-glucoside); df = dilution factor; 1 = path length in cm; ε = 26,900 M extinction coefficient in L mol^−1^ cm^−1^ for cyanidin 3-glucoside; 10^3^ = factor for conversion from grams to milligrams.

### 2.7. Antioxidant Activity Assays

#### 2.7.1. Ferric Reducing Antioxidant Power (FRAP)

The FRAP (ferric reducing antioxidant power) assay was determined according to Parra et al. [21]. In a 96-well microplate, 25 µL of stock solution was mixed with 175 µL of FRAP reagent. The mixture was incubated for 15 min at 37 °C, and absorption was recorded at 593 nm. The calibration curve was prepared with Trolox. Data were expressed as µmol TE/100 g FW.

#### 2.7.2. ABTS Method

The scavenging capacity assay towards ABTS^•+^ radicals was carried out according to the method of Echiburu-Chau et al. [22]. The radical ABTS solution was diluted with absolute ethanol until an initial absorbance of approximately 0.70 ± 0.03 was obtained at 734 nm. The radical discoloration was initiated by adding 20 µL of the stock solution to 200 µL of the ABTS^•+^ solution. After 7 min of incubation at 25 °C, the absorbance was measured at 734 nm. The calibration curve was prepared with Trolox. Data were expressed as µmol TE/100 g FW.

#### 2.7.3. Oxygen Radical Absorbance Capacity

The ORAC assay was performed according to Soto et al. with some modifications [20]. Fluorescein (8 nM), AAPH (75 mM), and a dilution of the sample extract were prepared in phosphate buffer solution (75 mM, pH 7.0). The sample (25 μL) was mixed with 100 µL of fluorescein in a Nunc 96-microwell black plates (Nunc, Rochester, NY, USA) and incubated at 37 °C for 30 min. Then, 75 µL of AAPH was added, and the fluorescein intensity was measured every minute for 120 min at excitation and emission wavelengths of 485 and 520 nm, respectively. The final ORAC values were calculated using the area under the curve (AUC) and the regression equation between Trolox and the net AUC. Data were expressed as µmol TE/100 g FW.

### 2.8. Antibacterial Activity

#### 2.8.1. Strain and Growth Conditions

*P. peruviana* extract was used to determine antibacterial activity against human pathogenic bacteria *Bacillus subtilis* (ATCC 6051), *Escherichia coli* (ATCC 23716), *Salmonella enterica* (ATCC 13311), *Pseudomonas aeruginosa* (ATCC 19429), and *Staphylococcus aureus* (ATCC 29737); and the phytopathogenic bacteria *Agrobacterium tumefaciens* (ATCC 19358), *Erwinia rhapontici* (MK883065), *Pantoea agglomerans* (MK883087), and *Pseudomonas syringae* (MF547632). Bacteria were inoculated into nutrient broth containing 5.0 g/L peptone and 3.0 g/L meat extract and incubated at 25 °C (plant pathogens) or 35 °C (human pathogens) for 18 h at 150 rpm using an incubator with orbital shaking LOM-80 (MRC Lab, London, UK).

#### 2.8.2. Minimum Inhibitory Concentration (MIC)

The minimum inhibitory concentration (MIC) of *P. peruviana* extract was determined using the method described by Simirgiotis et al. [24] to determine the minimum concentration necessary to inhibit bacterial growth. Working concentrations ranged from 0 to 20 mg/mL of fruit extract. Then, 40 mg/mL of extract stock solution was prepared to perform serial dilutions of *P. peruviana* extract and prepared in nutrient broth. Dilutions were prepared to a final working volume of 200 µL in 96-well plates and inoculated with the different bacteria to be tested at 25 °C or 35 °C, as appropriate. Dilutions of Cape gooseberry prepared in nutrient broth without bacteria were employed as a negative growth and sterility control. Nutrient broth without *P. peruviana* extract dilutions was inoculated with each bacterium and used as growth control. An assay using 0–90% (*v*/*v*) ethanol was performed to estimate the ethanol MIC for each bacterium. Concentrations higher than 20% ethanol were inhibitory for all bacteria. A solution of 40 mg/mL of fruit extract was prepared as a stock solution to avoid ethanol’s inhibitory activity during these assays. An additional assay was performed using 0–50 µg/mL of kanamycin, which was considered a positive control of bacterial growth inhibition. The MIC was determined from the lowest extract concentration, where no bacterial growth was observed after 24 h of incubation.

#### 2.8.3. Minimum Bactericidal Concentration (MBC)

The minimum bactericidal concentration (MBC) of Cape gooseberry extract was determined from the last three wells where no bacterial growth was observed in the MIC assay, as described by Simirgiotis et al. [24]. For this purpose, samples of 100 µL were taken from these wells and inoculated in nutrient broth plates supplemented with 1.5% agar. As a growth control, a culture without inhibition of the growth in the MIC test was used for each microorganism. Inoculated plates were incubated for 24 h at the corresponding temperature. The MBCs of *P. peruviana* extracts were determined from nutrient agar plates where no microbial growth was observed.

### 2.9. Inhibitory Activity Assays

#### 2.9.1. α-Amylase Inhibition Method

The α-amylase inhibition activity was evaluated according to the methodology described by Parra et al. [25]. For 5 min at 37 °C, 200 µL different concentrations of extract were incubated with 200 µL of starch at 1% *w*/*v*, and then 200 µL of α-amylase solution (10 U/mL) were added and incubated for a further 30 min. After the incubation, 400 µL of the color reagent was added and incubated for 15 min in boiling water. Hence, 40 µL of this mixture was diluted with 210 µL of water, and the absorbance at 565 nm was measured.

#### 2.9.2. α-Glucosidase Inhibition Method

The α-glucosidase inhibition assay was carried out as described by Parra et al. [25] with slight modifications. Briefly, 10 µL of 200 mM sodium phosphate buffer, 60 µL of different extract concentrations were prepared in the same buffer, and 10 µL of α-glucosidase (0.30 U/L) solution were mixed. After 15 min of preincubation at 37 °C, the reaction was started by adding 10 µL of p-nitrophenyl-α-d-glucopyranoside (1 mM). The reaction was further incubated for 30 min at 37 °C. Then, the absorbance at 415 nm was measured.

### 2.10. Statistical Analysis

The results are presented as the mean value of three determinations ± standard deviation. The Tukey comparison test determined significant differences between means (*p* values < 0.05 were regarded as significant). The originPro 9.1 software packages were used.

## 3. Results

### 3.1. Proximate Composition and Mineral Composition

The nutritional properties of Cape gooseberry are shown in Table 1. These values showed some non-significant differences with *P. peruviana* cultivated in the southern African region and Argentina [4,26]. This research showed that cultivated Cape gooseberry from the Andean region of the Atacama Desert favored the content of calcium, sodium, and phosphorus, compared to other reports for the same fruit grown in producing countries [3,4].

According to the obtained results, this fruit could provide an alternative for the daily intake of minerals (DIM) [4,28], considering a daily diet that incorporates 100 g of fresh fruit for an adult population. The content of K and Mg in 100 g of fruit provides more than 7% IDM of K and Mg and more than 10% IDM of Cu and P. These minerals have essential roles in human health. Concerning K, a high dietary intake protects people from conditions that affect the cardiovascular system, kidneys, and bones [28], whereas Cu is a critical mineral for the oxidative defense system and is needed to form hemoglobin [29]. Phosphorus plays a fundamental role in the maintenance of teeth and bones. Furthermore, it provides the body with the necessary resources to face wear and tear as a product of aging [28]. The potassium content found in samples was comparable to those of melons (267 mg/100 g FW), apricots, and pomegranates (259 mg/100 g FW) [30]. These samples were similar to berries in terms of magnesium content (20–22 mg/100 g FW), and the copper and phosphorus content was higher than that of many common fruits [30].

### 3.2. Chemical Composition

The phenolic profile of the ethanol extract of *Physalis peruviana* fruit was outlined by means of UHPLC-DAD analysis. A total of 12 phenolic compounds were detected. Table 2 shows the phenolic compounds identified in the extract of the *P. peruviana* fruit. The most abundant phenols were gallic acid (303.63 ± 35.85 mg/100 g DW), followed by 4-hydroxybenzoic acid (43.93 ± 3.45 mg/100 g DW) and kaempferol (19.57 ± 1.24 mg/100 g DW), whereas chlorogenic acid and apigenin were found in lower concentrations.

Regarding other studies of this fruit, *P. peruviana* grown in the Atacama Desert showed the highest gallic acid concentrations than those reported the same species grown in producing areas (183–180 mg/100 g DW) [13,31]. Gallic acid was the most abundant compound in all studies. The presence of this compound could be related to the control of hyperglycemia in diabetes attributed to the Cape gooseberry. Punithavathi et al. determined the effects of this compound in diabetic rats, finding that it positively altered carbohydrate metabolism and gluconeogenesis, increasing glycolysis, which decreased hyperglycemia [32]. Other studies reported that treating diabetic rats with gallic acid and p-coumaric acid significantly improved glucose tolerance, decreased oxidative stress in the brain, and improved antioxidant status [33]. Another main compound was 4-hydroxybenzoic acid, which has various functional biological properties, including antibacterial, anticancer, antidiabetic, antiaging, antiviral, and anti-inflammatory activities [34]. The content of this compound in the fruit grown in the Atacama was higher than that obtained for the same species (38.59 mg/100 g DW) [13] and the fruit of *Physalis pruinosa* (30.88 mg/100 g DW) [31] but was close to those reported for the fruit of *Elaeagnus angustifolia* (45.8 mg/100 g DW) [35], an original fruit from dry to semi-arid areas of Central Asia. Likewise, the kaempferol content found was double that reported for the same species [13] and higher than that reported for the Chilean species *Origanum vulgare* (5.76 mg/100 g DW), a species cultivated under the same conditions [25]. Some studies of diabetes show that kaempferol could be an antidiabetic agent and could be used as an adjuvant treatment for diabetes [36]. To our knowledge, no previous study has reported the presence of lutein in *P. peruviana*. Lutein is recognized for its anti-inflammatory and antioxidant properties [25]. The different concentrations of the compounds identified in this species depend on multiple factors, such as different extraction methods and experimental protocols and harvest time, growth stage, altitude, and climate [37]. In this context, the unique characteristics of the Cape gooseberry produced in this desertic growing area make the fruit an important natural source of phenolic compounds, as was proven for other species cultivated in desertic extreme habitats [38].

### 3.3. Bioactive Compounds and Antioxidant Capacity

The contents of phenols (26.24 mg GAE/100 g FW), flavonoids (1.48 mg QE/100 g FW), tannins (1.74 mg tannic acid/100 g FW), and anthocyanins (0.88 µg/100 g FW) present in the ethanolic extract of *P. peruviana* fruit from the Andean Region of the Atacama Desert are shown in Table 3. These results are similar to those reported by Yildiz et al. and El-Beltagi et al. [13,27], who studied fruits grown in Mediterranean climates. However, the phenol content of the fruit studied was higher than that reported for the same species grown under similar conditions (15.20 mg GAE/100 g FW) [4]. This value could be linked to the unique characteristics of northern Chile’s arid Andean region for the development of secondary metabolites [24,25]. Regarding the tannin content, the values are consistent with the flavor of the fruit observed, which shows slight astringency. Thus, the tannin value of this fruit was higher than that reported for the same fruit grown in Argentina [4].

Since a mixture of compounds determines the antioxidant activity of an extract, it is convenient to evaluate this property via more than one method [39]. Therefore, the method of individual electron transfer (SET) was used, such as FRAP and hydrogen atom abstraction (HAT) in ORAC. In the FRAP test, *P. peruviana* showed a reducing power below that determined for the standard quercetin (574.58 ± 56.50 µmol TE/100 g DW). However, the values of FRAP (69.58 ± 2.20 µmol TE/100 g FW) were higher than those obtained for the same species cultivated under similar conditions (11.12 µmol TE/100 g FW) [4] and close to those reported for some fruits of habitual consumption such as pears (64 ± 6 µmol TE/100 g FW) and watermelon (49 ± 4 µmol TE/100 g FW) [40]. The FRAP values were consistently higher than the ABTS values of antiradical activity, at 69.58 versus 24.99 µmol TE/100 g FW. This difference in values between methods could indicate that the antioxidant compounds present in Cape gooseberry are more reactive as reducers of ferric ions. The ORAC value for this sample was 3126.82 µmol TE/100 g fresh weight, and that value was below that reported for blueberries (5480–8750 µmol TE/100 g FW), but very similar to those reported for apricots, peaches, apples, and pears [41]. Differences in values across studies on this fruit could be attributed to differences in methodologies. Previous work has examined the ability of different assays, including ABTS, FRAP, and ORAC, to estimate antioxidant activity in other fruit extracts [42]. This study found that all four methods had comparable results when determining antioxidant activity but that the FRAP method had the highest reproducibility. Another potential cause for the difference in values is the difference in accession and ripeness among fruit samples. Bravo et al. [7] looked at 15 different accessions of Cape gooseberry at different ripeness levels and found that antioxidant activity was strongly influenced by the accession and maturity of the fruit.

### 3.4. Antibacterial Activity

The inhibitory and bactericidal activity of the *P. peruviana* fruit was tested against the human pathogenic bacteria *Escherichia coli*, *Pseudomonas aeruginosa*, *Salmonella enterica*, *Bacillus subtilis*, and *Staphylococcus aureus*, as well as the phytopathogenic bacteria *Erwinia rhapontici*, *Pseudomonas syringae*, *Pantoea agglomerans*, and *Agrobacterium tumefaciens* (Table 4).

The susceptibility to the *P. peruviana* extract of the different bacteria tested was variable and dependent on the bacterial species. In Table 4, it is possible to observe that almost all microorganisms were susceptible to the action of the extract of this fruit, with a variation in the MIC values from 0.63 mg/mL (*P. syringae*) to 5.00 mg/mL (*E. raphontici*) and MBC values of 5.00 mg/mL in all cases. This variation can be explained through specific differences in the composition of the different macromolecules and structures these bacteria possess. Kanamycin (50 µg) was used as a positive control of bacterial inhibition, showing different susceptibility according to the bacterial species. *E. rhapontici* was the most resistant bacterium (5.00 mg/mL MIC) and *P. syringae* was the most susceptible bacterium, of which the MIC was 0.63 mg/mL. These results are in agreement with those obtained by El-Beltagi et al. [13], Jaca and Kambizi [43], and Özgür et al. [44], who also showed the antibacterial activity of Cape gooseberry extracts against Gram-positive and Gram-negative bacteria. Different phytochemical compounds with the described antibacterial properties have been reported in *P. peruviana* extracts, which could explain the bacterial growth inhibition. Among the antimicrobial compounds present in *P. peruviana* fruit extract are flavonoids, phenols, and tannins. These molecules act through different mechanisms; for example, flavonoids are effective against several bacteria because they can form complex proteins (soluble and extracellular) and bacterial cell walls. Tannins form irreversible complexes with nucleophilic amino acids in proteins, activating proteins and promoting bacterial death [13]. Therefore, the antibacterial activity could be attributed to the concerted action of different phytochemical compounds in Cape gooseberry extracts. However, the results shown in Table 4 indicate low antibacterial activity in comparison to other vegetal extracts, such as methanolic fruit extracts of *Momordica charantia* [45] and fruit juice from *Citrus medica* Linn. [46], but comparable to some extracts of peel fruits of *Ecballium elaterium* [47]. In this context, these results open up an opportunity to continue research using higher concentrations or testing solvents of different polarities.

### 3.5. Enzyme Inhibitory Activity of Physalis peruviana

**Figure 2:**
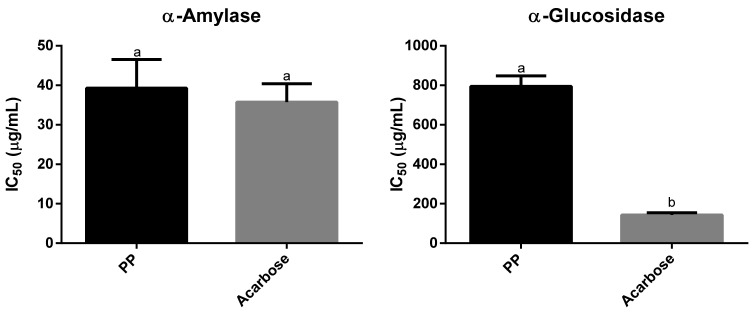
Enzyme inhibitory activities of *P. peruviana* extract. PP: *Phisalys peruviana*; acarbose positive control. Columns with different superscripts differ significantly (*p* < 0.05).

Hypertension, obesity, glycemic index imbalance, and glucose intolerance are early signs of the possible development of type 2 diabetes. An alternative to controlling hyperglycemia is to inhibit the key enzymes associated with type 2 diabetes, such as α-amylase and α-glucosidase [48]. An important strategy for controlling blood glucose levels is the inhibition of these enzymes. However, the main drawbacks of the inhibitors currently used are their side effects, such as diarrhea, bloating, and flatulence [49]. In the inhibition of α-amylase and α-glucosidase, the synergy of phenolic compounds could play a crucial role. In this work, the IC_50_ values of the ethanol extract of *P. peruviana* fruit on α-amylase and α-glucosidase were determined (Figure 2). The PP extract exerted a similar α-amylase inhibition (39.28 ± 7.25 µg/mL) to that of acarbose. Bernal et al. also reported that the α-amylase activity of *P. peruviana* fruit was similar to that determined for acarbose [9]. The inhibition of α-amylase did not show a significant difference compared to the reference standard, revealing that the extract of this fruit contains bioactive compounds that can inhibit α-amylase. The activity found for Cape gooseberry extract on α-amylase is higher than that reported for the extract of the same species (0.62 mg/mL) [12] and of medicinal plants used for the management of diabetes (*Aphloia theiformis*, IC_50_ 67.71 ± 1.21 µg/mL; *Andrographis paniculat*, IC_50_ 9.25 ± 0.12 mg/mL) [50,51].

On the other hand, compared to the positive control, this extract presented a low inhibitory activity on α-glucosidase (794.22 ± 53.42 µg/mL). However, it was higher than that reported by Rey et al. (4.11 mg/mL) [12] and Pinto et al. for the same species [52]. Although the Pinto report does not present IC_50_ values, it is possible to note that inhibitions close to 50% were achieved with concentrations higher than 10 mg/mL of this fruit extract. These results, along with the reported antidiabetic properties, indicate that this fruit has great potential as a source of compounds with hypoglycemic activity.

## 4. Conclusions

The current study demonstrates a series of in vitro activities for *P. peruviana* fruit adapted to northern Chile’s arid Andes’ climatic conditions. The findings demonstrate the excellent behavior of cultivated fruits in terms of their bioactive compounds (flavonoids and tannins) and antioxidant power compared to those reported in other production areas. This difference could be associated with the high solar radiation in this special desert environment that could directly influence the response of the secondary metabolites of the fruits. In addition, the mineral content found could constitute an alternative source for the daily intake of minerals. This species, cultivated in an arid area, can produce different secondary metabolites, while preserving the typical characteristics of the fruit. The chemical composition revealed that this fruit is a rich source of phenolic acids and flavonoids known for the control of hyperglycemia in diabetes, such as gallic acid and kaempferol. The activity found in this fruit showed a considerable inhibitory effect on the enzyme α-amylase, opening the door to the potential use of this fruit to manage chronic diseases such as diabetes. In summary, the cultivation of this fruit in extremely arid conditions stimulates the response of the metabolisms found for this species and enhances its bioactivity compared to the usual growing areas. In addition, this research has provided relevant information for food industries about *P. peruviana* grown in the arid region of the Chilean Andes and can be used to develop new products based on these fruits.

## Figures and Tables

**Table 1:** Nutritional properties of *P. peruviana* from the Arid Andean Region of Chile.

Proximate Composition	PP	Literature [4,26,27]
Water (g/100 g FW)	78.95 ± 2.01	73.77–78.9
Carbohydrates (g/100 g FW)	12.66 ± 0.04	12.91–19.66
Crude Protein (g/100 g FW)	1.43 ± 0.36	0.05–1.58
Crude Lipid (g/100 g FW)	0.20 ± 0.07	0.15–0.50
Crude Fiber (g/100 g FW	4.69 ± 0.10	4.12–4.90
Ash (g/100 g FW)	2.09 ± 0.68	0.77–2.95
**Mineral content**		
K (mg/100 g FW)	256.32 ± 2.20	362.03–373.25
Mg (mg/100 g FW)	20.04 ± 0.08	35.96–48.70
Ca (mg/100 g FW)	17.80 ± 0.12	8.00–11.17
Na (mg/100 g FW)	16.87 ± 0.22	8.41–8.78
P (mg/100 g FW)	94.75 ± 3.65	55.3
Mn (mg/100 g FW)	0.17 ± 0.01	--
Zn (mg/100 g FW)	0.15 ± 0.05	--
Cu (mg/100 g FW)	0.09 ± 0.01	0.26–0.35
Fe (mg/100 g FW)	0.54 ± 0.05	1.2

Values are shown as mean ± standard deviations; FW: fresh weight; PP: *Physalis peruviana.*

**Table 2:**
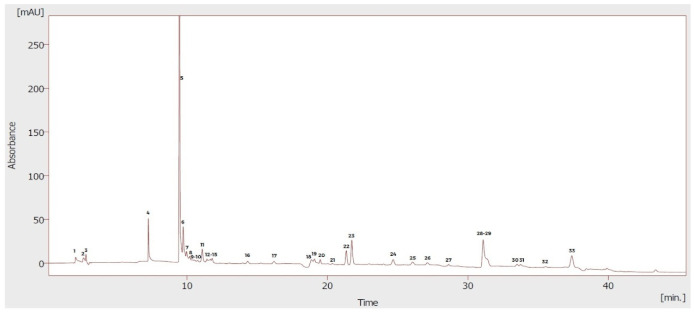
The phenolic compounds identified in the extract of the fruit of *P. peruviana*.

Peak	Phenolic Compounds	RT	PP *
5	Gallic acid	9.46	303.63 ± 35.85
6	4-hydroxybenzoic acid	9.73	43.93 ± 3.45
7	Caffeic acid	9.97	15.16 ± 2.22
8	Vanillic acid	10.20	9.05 ± 1.11
10	Chlorogenic acid	10.74	1.25 ± 0.07
11	Ferulic acid	11.08	15.89 ± 0.75
12	*p*-Coumaric acid	11.42	4.29 ± 0.31
16	*trans*-Cinnamic acid	14.32	4.09 ± 0.14
18	Luteolin	18.85	15.17 ± 1.85
20	Naringenin	19.48	9.47 ± 0.49
21	Apigenin	20.34	1.85 ± 0.02
22	Kaempferol	21.34	19.57 ± 1.24

* (mg/100 g of DW); values are shown as mean ± standard deviations; RT: retention time; PP: *Physalis peruviana.* Only the compounds described in the table above were identified.

**Table 3:** Phytochemical analysis and antioxidant activity of *P. peruviana*.

Phytochemical Analysis	PP	Literature [4,13,27]
Total Polyphenol (mg GAE/100 g FW)	26.24 ± 2.16	27.32–7.10
Total Flavonoids (mg QE/100 g FW)	1.48 ± 0.04	1.28
Total tannins (mg Tannic acid/100 g FW)	1.74 ± 0.33	1.10–0.60
Total anthocyanins (µg/100 g FW)	0.88 ± 0.02	1.34
**Antioxidant Activity**		
FRAP (µmol TE/100 g FW)	69.58 ± 2.20	11.12–8.96
ABTS (µmol TE/100 g FW)	24.99 ± 1.15	3.76–2.60
ORAC (µmol TE/100 g FW)	3126.82 ± 30.68	--

Values are shown as mean ± standard deviations; FW: fresh weight; PP: *Physalis peruviana.*

**Table 4:** Antibacterial activity of *P. peruviana* from the Arid Andean Region of Chile.

Bacterium	MIC ^1^ (mg/mL)	MIC Kan * (µg/mL)	MBC ^2^ (mg/mL)	MBC Kan * (µg/mL)
*Bacillus subtilis* (ATCC 6051)	2.50	1.25	5.00	20.00
*Escherichia coli* (ATCC 23716)	2.50	5.00	5.00	10.00
*Pseudomonas aeruginosa* (ATCC 19429)	2.50	5.00	5.00	10.00
*Salmonella enterica* (ATCC 13311)	1.25	2.50	5.00	5.00
*Staphylococcus aureus* (ATCC 29737)	1.25	2.50	5.00	10.00
*Agrobacterium tumefaciens* (ATCC 19358)	1.25	1.25	5.00	5.00
*Erwinia rhapontici* (MK883065)	5.00	1.25	5.00	2.50
*Pantoea agglomerans* (MK883087)	1.25	2.50	5.00	5.00
*Pseudomonas syringae* (MF547632)	0.63	1.25	5.00	2.50

^1^ Minimum inhibitory concentration. ^2^ Minimum bactericidal concentration. * Kan: Kanamycin positive control. ATCC: American Type Culture Collection (USA). MK883065, MF547632, MK883087, and MH885473 are the accession numbers in Genbank of the respective bacteria.

## Data Availability

Data are contained within the article.
